# Gut microbiota and metabolite features in NSCLC nude mouse models of subcutaneous tumor and leptomeningeal metastasis: a microbiome-metabolome combined analysis

**DOI:** 10.3389/fcimb.2025.1616695

**Published:** 2025-06-27

**Authors:** Yang Du, Chengjuan Fan, Xiaowei Song, Chong Teng, Zhichao Zhang, Jing Zhang, Tianjiao Zhao, Tao Xin

**Affiliations:** Department of Oncology, the Second Affiliated Hospital of Harbin Medical University, Harbin, China

**Keywords:** lung cancer, leptomeningeal metastasis, gut microbiota, metabolite lung cancer, metabolite

## Abstract

**Background:**

The incidence and mortality rates of lung cancer are both elevated. In lung cancer, leptomeningeal metastasis (LM) is a serious consequence. Patients suffering from LM have severe symptoms and a short survival time. Numerous studies have shown a connection between the prognosis of lung cancer and the composition of the gut microbiota. However, Current knowledge regarding the gut microbiota and metabolites in lung cancer patients with LM, as well as their potential impacts on LM pathogenesis, remains remarkably limited.

**Method:**

We established a mouse model of LM from lung cancer and a subcutaneous metastatic model, using wild-type mice as controls. Each of the three groups above contained six mice. We examined the fecal microbiota and metabolites of three groups of mice utilizing 16S rRNA gene sequencing and liquid chromatography-mass spectrometry (LC-MS) technologies. Conducting correlation analysis on microbiome and metabolome data concurrently to identify significant relationships.

**Result:**

Mice with LM had a different gut microbiota and metabolite composition than wild-type and subcutaneous metastatic mice; the LM group had a higher ratio of Firmicutes to Bacteroidetes. Differential metabolites are primarily seen in pathways such as Nicotinate and nicotinamide metabolism, Tryptophan metabolism; Association analysis reveals that some changes in gut microbiota are linked to metabolites, such as a positive association between Eubacteria and N-Acetylsorotonin.

**Conclusion:**

Some microbiota and metabolites may act as biomarkers for LM, controlling gut microbiota and metabolites or giving a novel option for research into lung cancer leptomeningeal metastases.

## Introduction

Lung cancer is among the most prevalent malignant neoplasms, distinguished by the greatest incidence and fatality rates globally. Lung cancer is categorized into two primary types: non-small cell lung cancer (NSCLC) and small cell lung cancer. NSCLC constitutes almost 85% of cases. Leptomeningeal metastasis (LM) is a severe complication of NSCLC, characterized by significant symptoms and the worst prognosis. The prevalence of leptomeningeal metastases in NSCLC can range from 9% to 26% (Mack et al., 2016; Thakkar et al., 2020). Because the blood-brain barrier (BBB) blocks most therapeutics, NSCLC patients with leptomeningeal metastases have a median overall survival of only 3.6–11 months (Umemura et al., 2011; Liao et al., 2015). Our center specializes in diagnosing and treating lung cancer with leptomeningeal metastases. We intend to investigate gut microbiota and metabolome attributes by developing a mouse model of leptomeningeal metastases and screening for potential biomarkers.

Emerging evidence highlights associations between gut microbiota and disease diagnosis, therapeutic response, and clinical outcomes. The gut microbiome can influence human health and affect physiology through its influence on pathogen resistance, gut barrier maintenance, metabolism, immunity, and neural signaling. In particular, gut–brain and gutliver axes have already been investigated, while the relation between gut–lung axis has been newly suggested (Anand and Mande, 2018), in particular the hypothesis that changes in the gut microbiota could influence the lung microbiota, and vice versa (Botticelli et al., 2020).

Numerous investigations have demonstrated the connection between gut microbiota and hematological cancers, lung cancer, and colorectal cancer (Nawfal et al., 2023; Peppas et al., 2023; Delzenne et al., 2024). Despite growing evidence of gut microbiota’s role in primary lung cancer, research on its association with LM remains relatively insufficient. Studies have shown that the gut microbiota can influence neurogenesis, myelination, dendritic morphology, microglia morphology, BBB structure and permeability, synapse structure and function (Yassin et al., 2025). Studies have shown that supplementing mice with Lactobacillus plantarum can enhance the integrity of the BBB (Dhaliwal et al., 2018).Supplementation with Clostridium butyricum can reduce neuronal damage and increase BBB permeability (Li et al., 2017). Some gut bacterial metabolites, such as butyric acid and propionic acid, enhance the integrity of the epithelial barrier by promoting intercellular tight junctions (Peng et al., 2009; Tong et al., 2016). So we believe that there may be a correlation between gut microbiota and the occurrence and development of LM.

Additionally, research indicates that the gut microbiota may serve as a biomarker for the effectiveness of tumor immunotherapy (Ning and Hong, 2024). Zheng et al. created a predictive model based on operational taxonomic units (OTUs) for early lung cancer diagnosis by sequencing, even impacting the treatment of cancers and disorders (Zheng et al., 2020). However, gut microbiota differences alone cannot fully explain their mechanistic link to cancer progression or downstream metabolic effects (Botticelli et al., 2020). Notably, gut microbiota-derived metabolites (e.g., short-chain fatty acids, bile acids) can systemically influence tumor metabolism and immune responses, suggesting potential crosstalk between microbial and metabolic signatures in leptomeningeal metastasis.

Metabolism plays a key role in cancer initiation and progression. One of the earliest recognized metabolic changes is the increased glucose uptake by tumors (Schmidt et al., 2021). Cancer and cancer therapies can also alter metabolism at the whole-body level and interact with the metabolic effects of diet and exercise in complex ways that may affect cancer outcomes and impact a patient’s quality of life (Schmidt et al., 2021). In recent years, there has been a renewed interest in understanding how altered metabolism contributes to cancer pathogenesis. Many factors, such as tumor hypoxia, stromal composition, immune cell infiltration, and genetic alterations, play critical roles in defining cancer cell metabolism. Thus, we integrated metabolomics to examine the features of leptomeningeal metastases from several angles, based on investigating gut microbiota.

We established leptomeningeal metastasis and subcutaneous tumor models using BALB/c nude mice, with wild-type mice as controls. Fecal samples were collected from three groups of mice for microbial and metabolomic sequencing to investigate the distinctive microorganisms and metabolites associated with lung cancer meningeal metastasis in comparison to wild-type and subcutaneous tumor mice.

## Materials and methods

### Construction of tumor models and sample collection

18 SPF-grade 7-week-old BALB/c nude mice (Liaoning Changsheng Biotechnology) were randomly allocated into three groups (n=6/group, 3 males and 3 females per group): healthy controls (N), subcutaneous tumor models (P), and LM models. PC9 human lung adenocarcinoma cells cultured in RPMI-1640 with 10% FBS were harvested at 1×10^7^ cells/mL for subcutaneous injection (200 μL) into the right axillary region under 2% isoflurane anesthesia, with successful modeling confirmed by palpable tumors (>100 mm³) within 1 week. For the LM model, luciferase-labeled PC9 cells (1 × 10^6^ cells/ml) were injected into the cerebellomedullary cistern (10 μL). At 5 days post-injection, bioluminescence signals were detected by a small animal real-time imaging system (PerkinElmer, USA), confirming successful tumor implantation. After confirming the successful establishment of the model, fresh feces excreted by mice were collected at 19:00 (1 hour after the end of the daily light cycle). To induce defecation, sterile cotton swabs were gently pressed on the mice’s lower abdomen along the intestinal path. Fresh fecal samples (approximately 200 mg/mouse) were then collected, immediately flash-frozen in liquid nitrogen, and stored at -80°C until further processing. The animal experimental part of this study has been approved by the Ethics Committee of the Second Affiliated Hospital of Harbin Medical University. Ethical approval number: YJSDW2024-125.

### Microbial DNA extraction and sequencing

Total genomic DNA was extracted from fecal samples using the TGuide S96 Magnetic Bead DNA Extraction Kit (Tiangen Biotech, Beijing, China) following the manufacturer’s protocol. DNA concentration and purity were determined using a NanoDrop 2000 spectrophotometer (Thermo Fisher Scientific, USA), while integrity was verified by 1% agarose gel electrophoresis. The hypervariable V3-V4 region of bacterial 16S rRNA gene was amplified using universal primers (5’-ACTCCTACGGGAGGCAGCA-3’) and (5’-GGACTACHVGGGTWTCTAAT-3’). PCR products were purified using the OMEGA DNA Clean-Up Kit and subsequently recovered by gel extraction with the Monarch DNA Gel Extraction Kit. Finally, paired-end sequencing was performed on the Illumina NovaSeq 6000 platform according to standard protocols.

### 16S rRNA sequencing result data processing

Concatenate the raw data (FLASH, v1.2.11), filter the quality of the concatenated sequence (Trimmomatic, v0.33), apply the DADA2 method in QIIME2 for denoising, concatenate the paired-end sequences, and eliminate sequences with ambiguous bases, single base homologous regions, and chimeras to ensure precise impurity removal and result accuracy (Magoč and Salzberg, 2011).

Cluster the sequences at a 97% similarity threshold and categorize them according to operational taxonomic units (OTUs) (USEARCH, v10.0). Employing 0.005% of the total sequencing sequences as a criterion to filter OTUs, each deduplicated sequence produced following DADA2 quality control is designated as Amplification Sequence Variants (ASVs).

The VennDiagram package in R software was utilized to compute and illustrate Venn diagrams, effectively presenting the distinct and common numbers of microorganisms across the three groups, so intuitively depicting the overlap of characteristics among the samples.

Microbial community composition was analyzed at multiple taxonomic levels (phylum, family, and genus) using QIIME2, with results visualized as stacked bar plots. Alpha diversity (Chao1 richness and Shannon diversity indices) was calculated in QIIME2 and plotted using ggplot2 (R). Beta diversity was assessed based on Bray-Curtis dissimilarity matrices, visualized through principal coordinates analysis (PCoA) using the ape package (R). Linear discriminant analysis effect size (LEfSe) was performed using the Python LEfSe package to identify differentially abundant taxa between groups. Only taxa meeting both criteria (p<0.05 and log10 LDA score ≥3.5) were considered statistically significant.

### LC/MS non-targeted metabolomics analysis

Firstly, extract metabolites, The main steps for metabolite extraction include adding an appropriate volume of extraction solution and magnetic beads for grinding and sonication. After centrifugation and collection of the supernatant, vacuum drying is performed. Subsequently, an appropriate amount of extraction solution is added for reconstitution before analysis on the instrument.

The LC/MS system for metabolomics analysis is composed of Waters Acquity I-Class PLUS ultra-high performance liquid tandem Waters Xevo G2-XS QTof high resolution mass spectrometer. The column used is purchased from Waters Acquity UPLC HSS T3 column (1.8um 2.1*100mm). Positive ion mode: mobile phase A: 0.1% formic acid aqueous solution; mobile phase B: 0.1% formic acid acetonitrile. Negative ion mode: mobile phase A: 0.1% formic acid aqueous solution; mobile phase B: 0.1% formic acid acetonitrile.

The raw data collected using MassLynx V4.2 was processed using Progenesis QI software for peak extraction, peak alignment, and other data processing operations. Identification is performed using the Progenesis QI software with the online METLIN database, public databases, and a self-built database. Theoretical fragment identification is also conducted.

After metabolite qualitative and quantitative analysis, data quality assessment, annotation analysis, differential expression analysis, and functional enrichment are performed.

### Statistical analysis

SPSS software (v 26.0) and GraphPad Prism software (v 9.2.0) Statistical analysis and visualization of data using R-studio software (v 3.3.1) Chemical processing was performed using QIIME (v 1.6.0) for bioinformatics analysis. Student’s t-test and Fisher’s exact test were used to compare sample baseline data. Adonis analysis, Wilcoxon ranksum test, and Kruskal–Wallis test were used to compare the differences between microbial groups. The metabolomics data were processed and analyzed using the Progenesis QI v2.3 software. Student’s t-test and fold change analysis were used to compare metabolites between groups. Pearson correlation coefficient was used to measure the degree of linear correlation between two metabolites. The Spearman rank correlation test was used to assess the correlation between microorganisms and metabolites. p<0.05 was considered as statistically significant.

## Result

### Microbial characteristics of different groups

**Figure 1:**
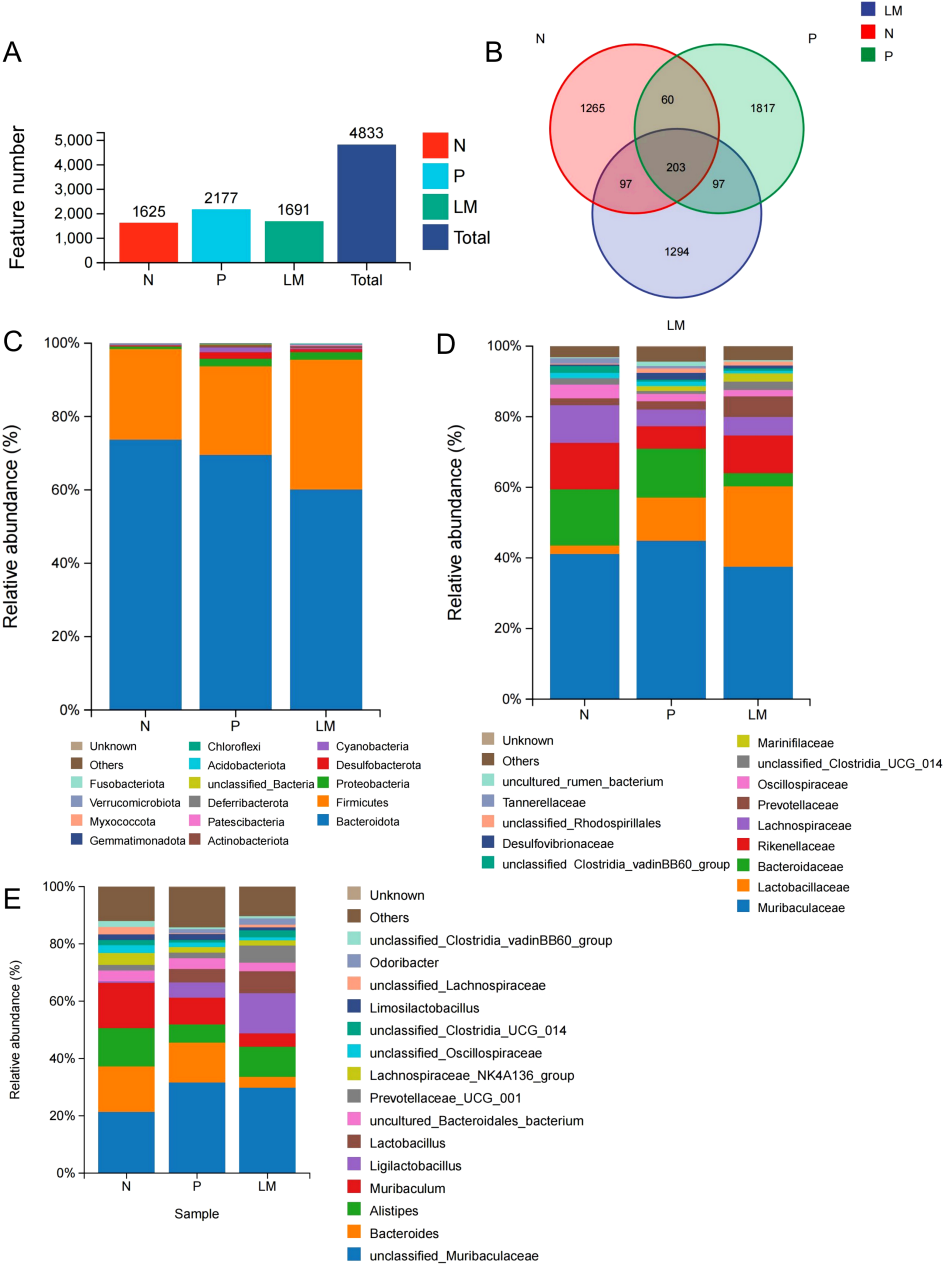
Intestinal flora community structure. **(A)** Number of OTUs in different groups. **(B)** The Venn diagram showed each group of unique and common OTUs. **(C-E)** At the level of phylum **(C)**, species **(D)**, and genus **(E)**, the top 15 representative species and their proportion in the three groups.

Eighteen fecal samples were collected, comprising six samples from each of the three groups: leptomeningeal metastasis (LM), subcutaneous tumor (P), and control (N). The sequencing results revealed 4833 unique OTUs, with 1625, 2177, and 1691 identified in the N, P, and LM groups, respectively (**Figure 1A**). We illustrated the shared and distinct OTUs of microbial communities among three groups using a Venn diagram (**Figure 1B**).

The microbial community structure was analyzed in each group, and the top 15 microbiota with the highest proportions were identified based on their diversity. Bacteroidetes and Firmicutes represent the predominant phyla across the three groups. The tumor group shows increased levels of Proteobacteria and Desulfobacter relative to the N group, as demonstrated in **Figure 1C**.

At the family level, Muribaculaceae showed similar abundance across all groups, while Lactobacillus was more abundant in the tumor group compared to the N group (2.4%) and was also higher in the LM group (22.8%) than in the P group (12.2%). The control group demonstrated a greater abundance of Bacteroidaceae, Rikenellaceae, and Lachnospiraceae in comparison to the LM and P groups. The N:P:LM ratio for Bacteroidaceae was 15.9:14.0:3.8, while for Rikenellaceae it was 13.3:6.4:10.7. The N:P:LM ratio for Lachnospiraceae is 10.6:4.8:5.3. (**Figure 1D**). At the genus level, Ligullacoccus (14.0%) and Alistipes (10.6%) are the dominant bacterial genera in the LM group, whereas Bacteroides (14.0%) and Muribaculum (9.4%) are the most prevalent in the P group. Group N displays the highest abundance of Muribaculum (16.0%), Bacteroides (15.9%), and Alistipes (13.3%). (**Figure 1E**).

**Figure 2:**
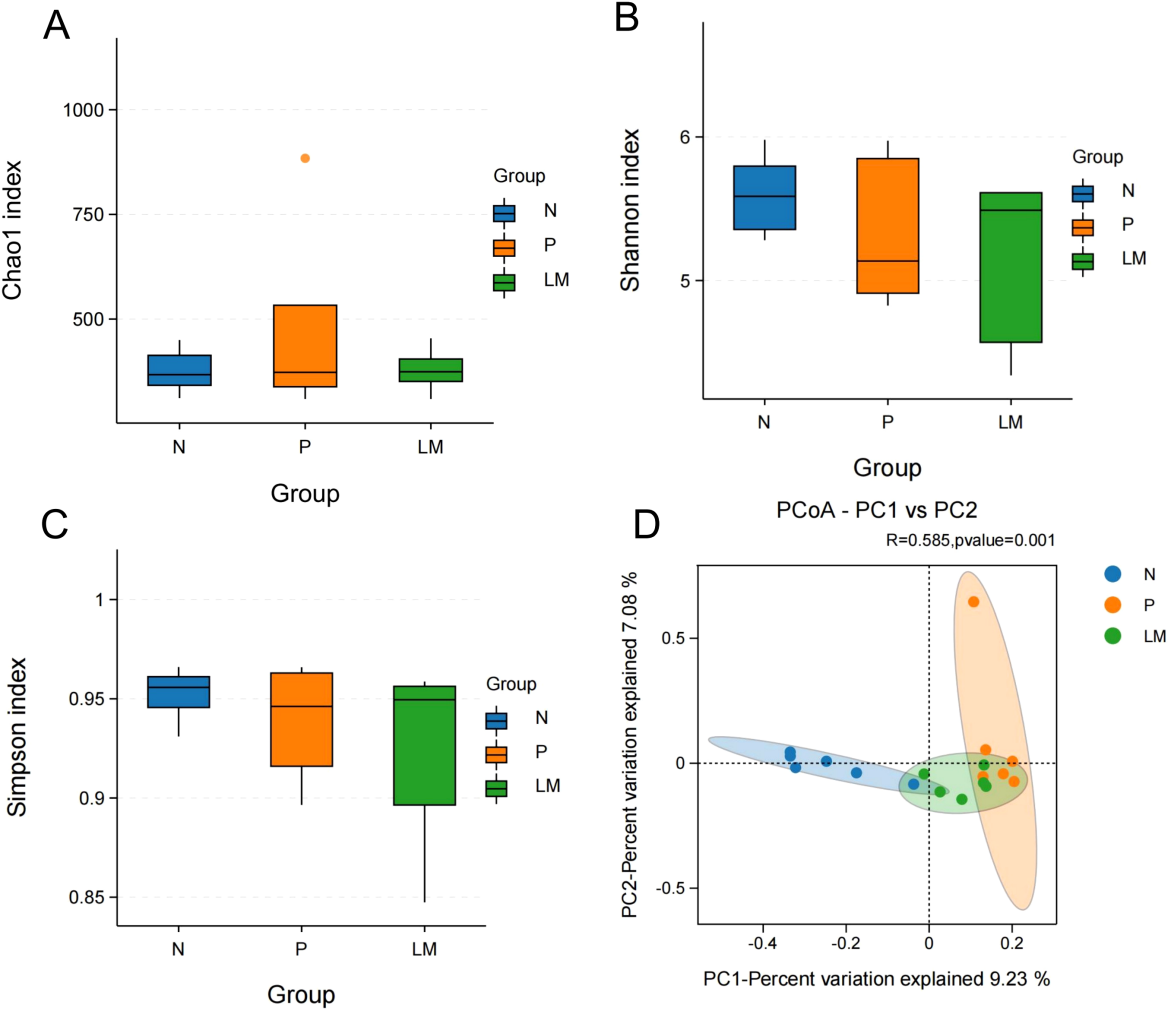
Alpha diversity and beta diversity. **(A)** Chao1 index between three groups. **(B)** Shannon index between three groups. **(C)** Simpson index between three groups. **(D)** PCoA shows differences between individuals or groups. The abscissa (PC1) and the ordinate (PC2) are the two main coordinates that explain the greatest difference between samples.

Alpha diversity reflects the richness and variety of species present within individual samples. The Chao1 index measures species richness and counts species, while the Shannon and Simpson indices assess species diversity. The Chao1 index indicates no significant differences in community richness between the groups. The Shannon and Simpson indices indicate variations in species diversity among the groups; however, these differences lack statistical significance, as shown in **Figures 2A-C**.

Beta diversity was used to examine the variations in the composition of different gut microbiota communities. This research assessed beta diversity using PCoA based on the Bray-Curtis distance matrix, with inter-group differences examined through PERMANOVA. The results demonstrated significant differences among the three groups, with statistical significance achieved (P = 0.001), as shown in **Figure 2D**.

### Analysis of differences in intestinal microbiota

**Figure 3:**
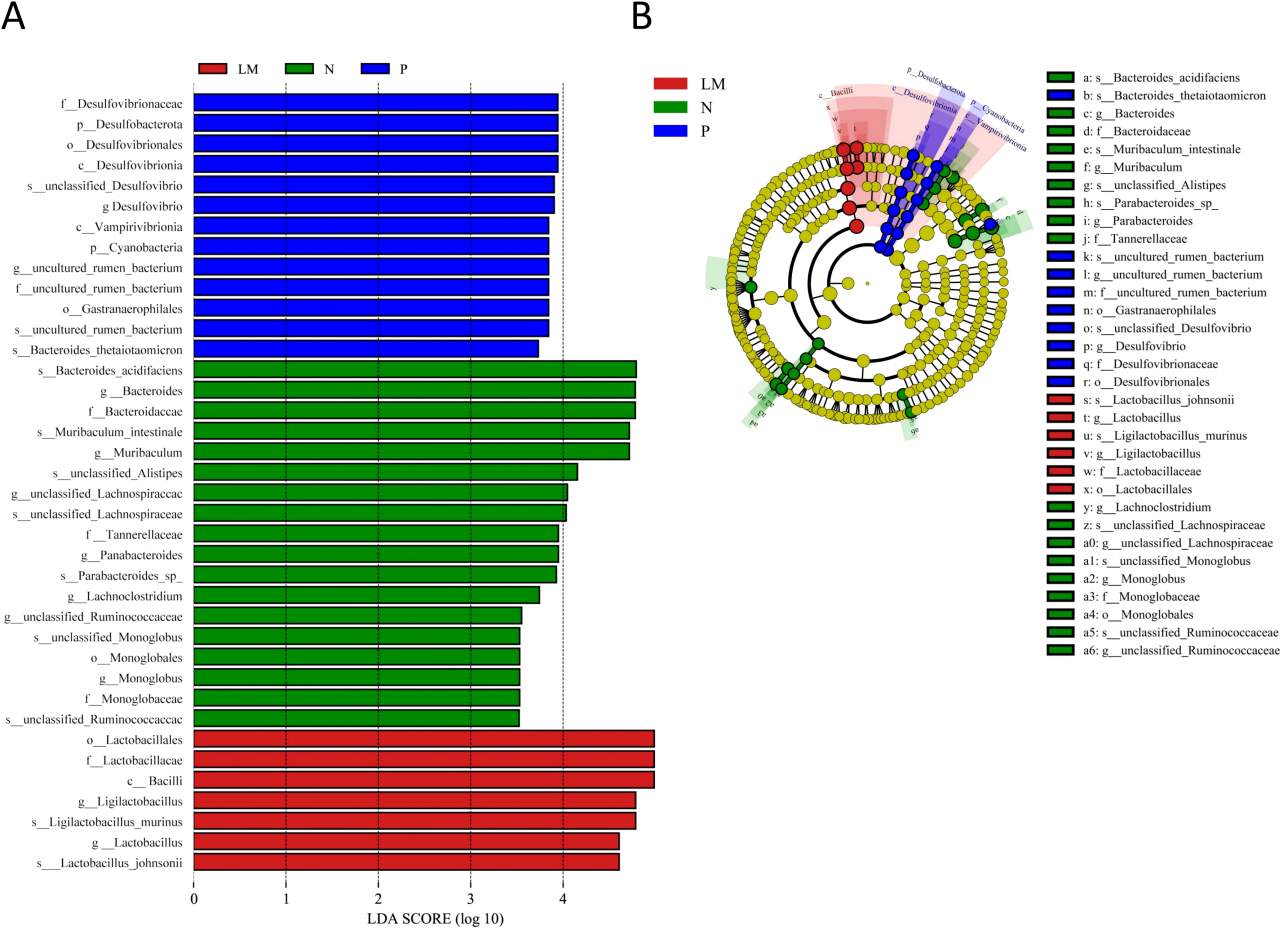
LEfSe analysis. **(A)** Histogram of LDA value distribution. **(B)** Cladogram.

Linear discriminant analysis (LDA) effect size (LEfSe) was used to identify key microbial taxa. When the LDA score surpasses 3.5, a total of 38 species exhibit significant differences among the three groups. The groups N, P, and LM were characterized by seven, two, and two distinct genera and species, respectively. Group N: Bacteroides (LDA=4.79, P=0.011), Muribaculum (LDA=4.72, P=0.042), unclassified Lachnospiraceae (LDA=4.03, P=0.010), Parabacteroides (LDA=3.93, P=0.015), Lachnoclostridium (LDA=3.74, P=0.045), unclassified Ruminococcaceae (LDA=3.52, P=0.005), Monoglobus (L Group P: Desulfovibrio (LDA=3.90, P=0.006), uncultured rumen bacterium (LDA=3.84, P=0.017). Group LM: Ligilactobacillus (LDA=4.79, P=0.026), Lactobacillus (LDA=4.61, P=0.005). Refer to **Figure 3**.

### Analysis of differential metabolites across three groups

**Figure 4:**
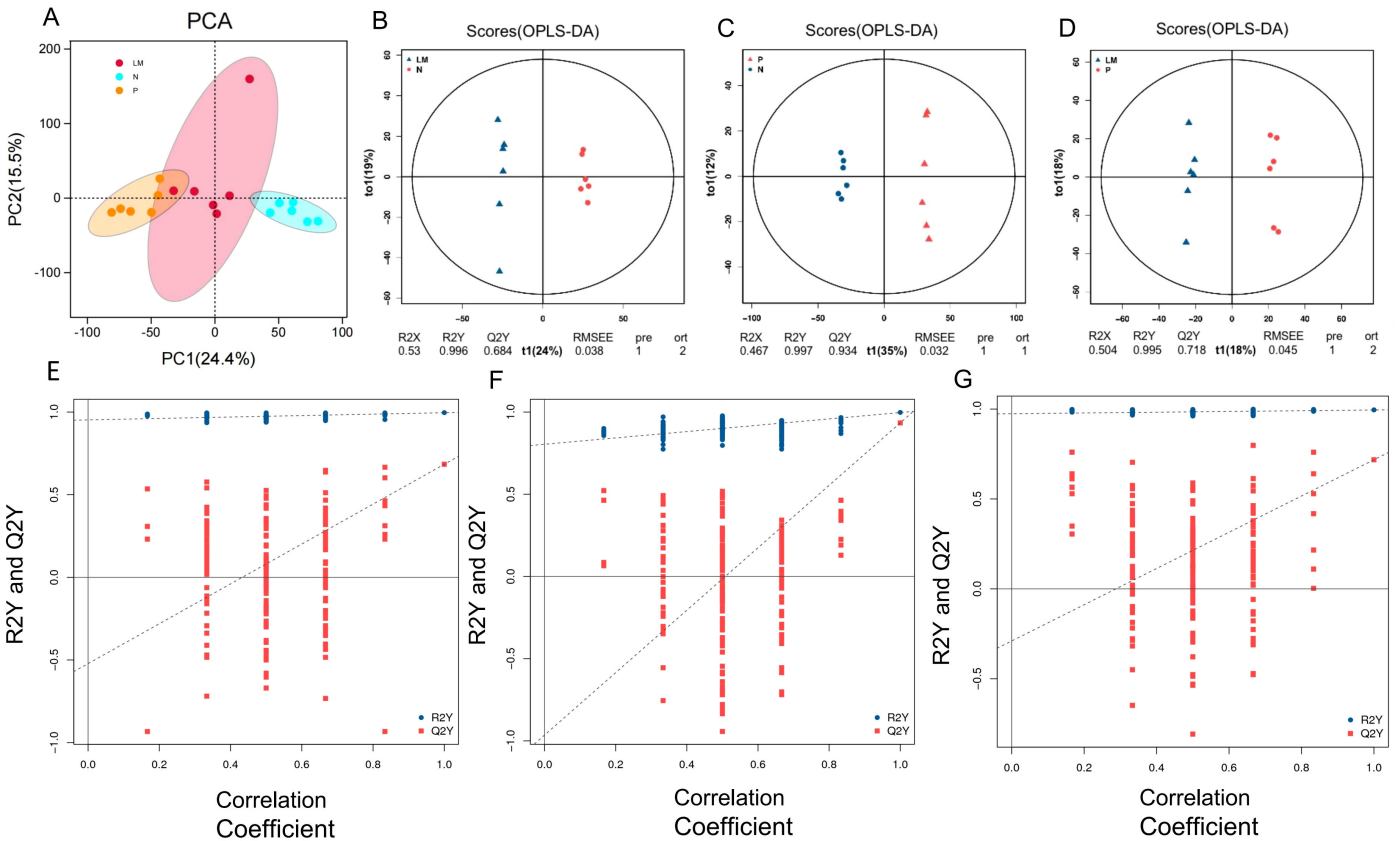
Principal component analysis. **(A)** Score plot of the PCA model in three models. **(B-D)** OPLS-DA score chart shows the difference in metabolites between groups. The abscissa represents the variation between groups, and the ordinate represents the variation within groups. **(E-G)** Comparison of the true model parameters in the validation test and those of permutated models. **(B, E)** LM vs N. **(C, F)** P vs N. **(D, G)** LM vs P.

This study was based on the LC-QTOF platform and involved metabolomics qualitative and quantitative analysis of 18 samples. By performing PCA on samples, it is possible to gain preliminary insights into the overall metabolic differences among sample groups and the variability within each group (**Figure 4A**). Based on the variable importance (VIP) in the projection, filter criteria are implemented using FC=2, P=0.05, and VIP=1 as threshold parameters to screen out differential metabolites between each two groups. The results are shown in **Table 1**.

**Table 1:** The number of differential metabolites in each group.

Group	DEMs_total	DEMs_up	DEMs_down
LM vs N	452	226	226
P vs N	947	526	421
LM vs P	282	139	143

(FC=2 Pvalue=0.05 VIP=1).

The significant abundance of metabolites identified via non-target metabolomics requires the application of orthogonal projections to latent structures discriminant analysis (OPLS-DA) to enhance the extraction of information from sequencing results. The results demonstrate notable variations in metabolic processes among different groups. Refer to **Figures 4B-D**.

To assess the reliability of the OPLS-DA model, a permutation test is conducted. In this test, the sample groups are randomly shuffled, and the OPLS-DA model is built using the permuted groups. The R2Y and Q2Y values are calculated for each permutation. This process is repeated multiple times. If the slope of the Q2Y regression line is positive, it indicates a meaningful model. If the blue dots are generally located above the red dots, it suggests good independence between the modeling training set and the testing set. (**Figures 4E-G**).

**Figure 5:**
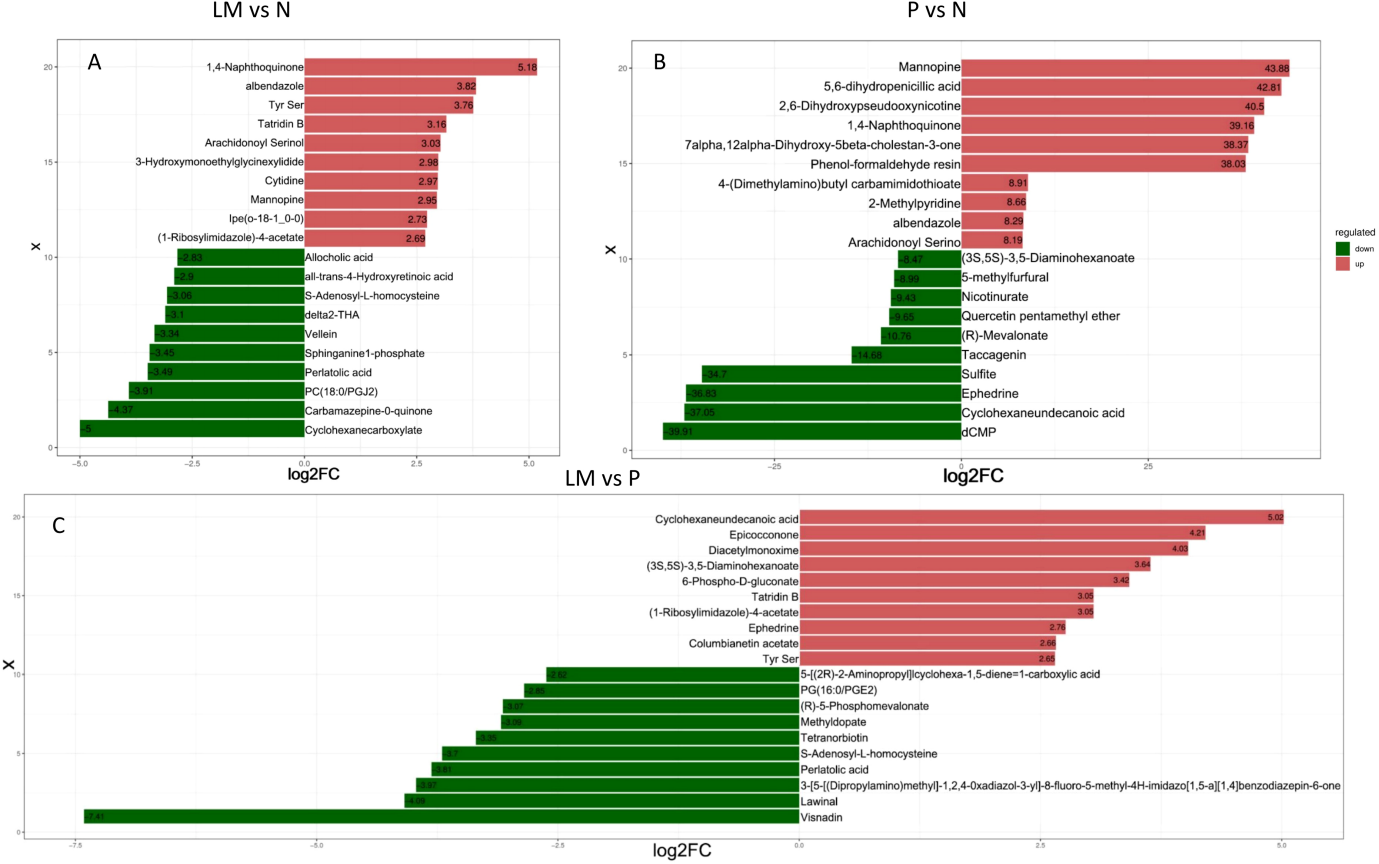
LogFC results of upregulation and downregulation of the top 10 metabolites in the experimental group compared to the control group. **(A)** LM vs N. **(B)** P vs N. **(C)** LM vs P.

Subsequently, we will analyze the fold change variations in the quantitative data of metabolites across each group. The three sets of data will be compared pairwise, and the logFC results for the top 10 upregulated and downregulated metabolites will be presented, as illustrated in **Figure 5**.

**Figure 6:**
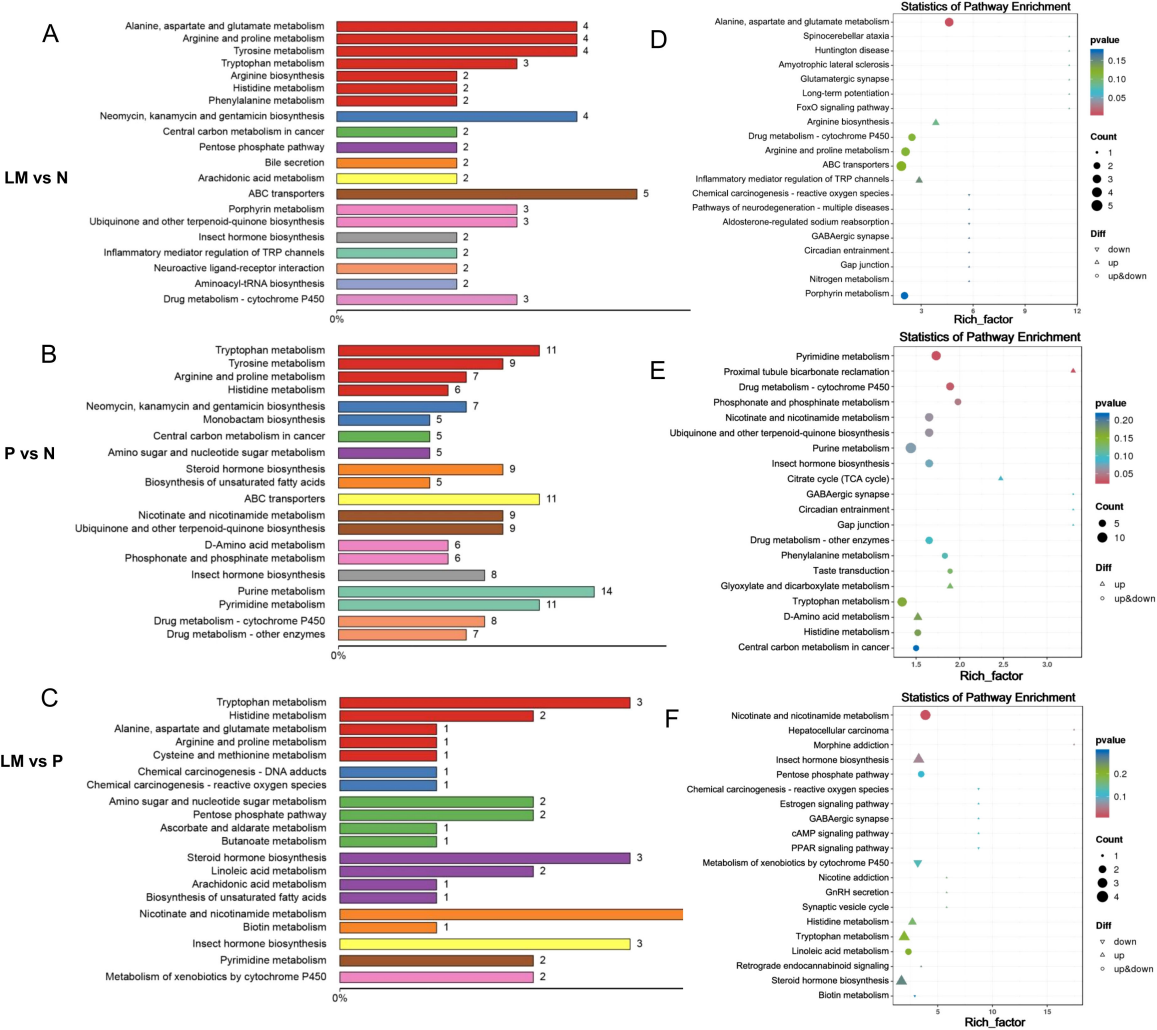
Enriched differential metabolites in the KEGG pathway. **(A-C)** The proportion of differentially expressed metabolites annotated by various KEGG pathways is illustrated in a bar chart. **(D-F)** The enrichment factor is represented by the horizontal axis, while the name of the metabolic pathway is represented by the vertical axis. The enrichment factor is the ratio of differential metabolites annotated to a specific pathway among differential metabolites to the ratio of metabolites annotated to that pathway among all metabolites.

Metabolites interact with each other in biological systems, forming different pathways. Annotate differential metabolites using the KEGG database, select the top 20 entries with the most annotated differential metabolites in the pathway, and draw a summary bar chart and point graph. **Figure 6**.

**Figure 7:**
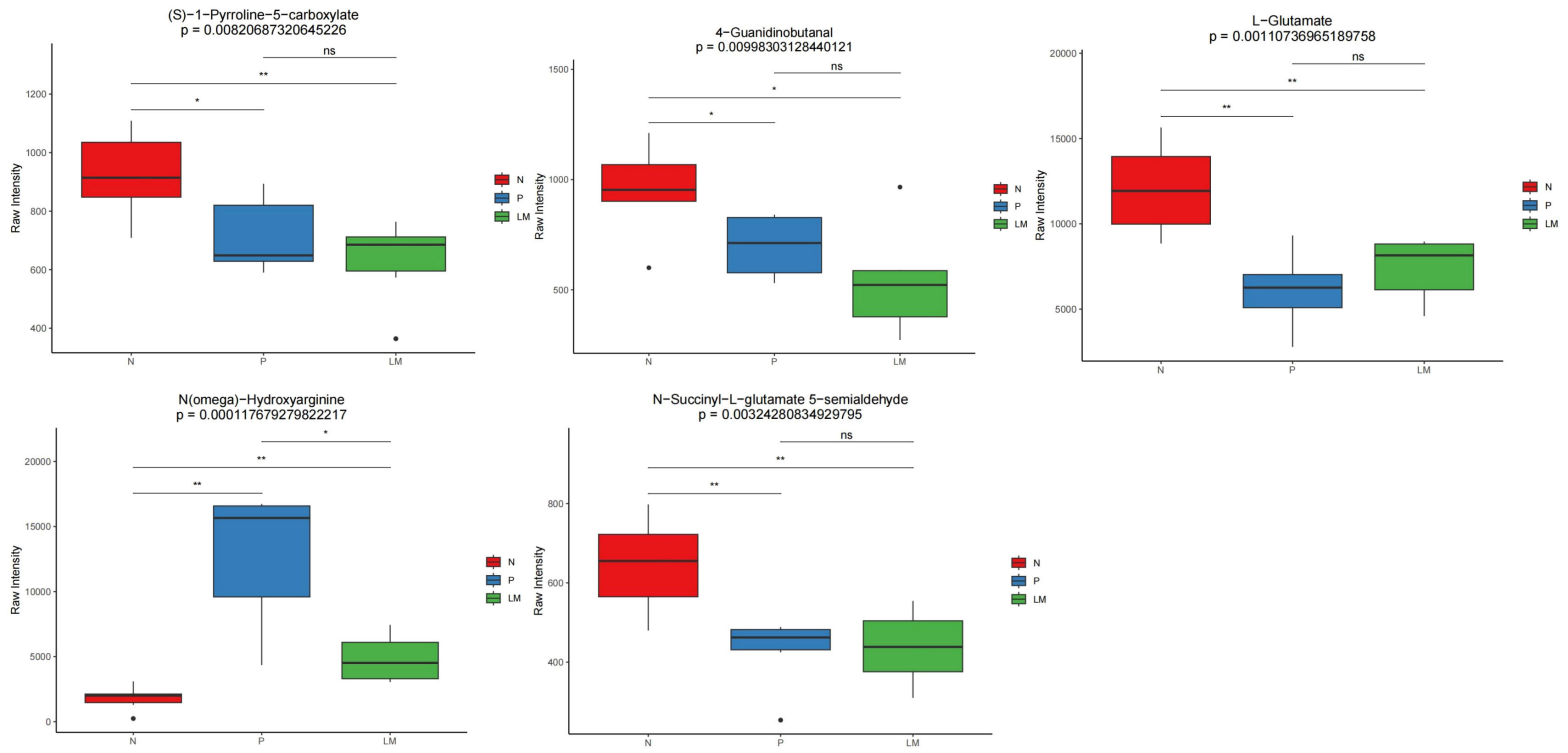
Distribution of differential metabolites related to Arginine and proline metabolism pathway in each group. *p < 0.05, **p < 0.01, ns: not signisicent, p ≥ 0.05.

The pathways predominantly enriched by the differential metabolites in pairwise comparisons are as follows: LM vs N: ABC transporters, Alanine, aspartate and glutamate metabolism, Arginine and proline metabolism, Tyrosine metabolism, Neomycin, kanamycin and gentamicin biosynthesis. P vs N: Purine metabolism, Pyrimidine metabolism, Tryptophan metabolism, ABC transporters. LM vs P: Nicotinate and nicotinamide metabolism, Insect hormone biosynthesis, Tryptophan metabolism. Among them, the pathway ABC transporters, Alanine, aspartate and glutamate metabolism, Arginine and proline metabolism, Tryptophan metabolism, Purine metabolism and Pyrimidine metabolism are associated with the occurrence and development of lung cancer. Previous studies have shown that the Arginine and proline metabolism pathway is closely related to the development of tumors (Wang et al., 2024). We will compare the metabolites involved in this metabolic pathway. Refer to **Figure 7**. The metabolite L-Glutamate in this pathway was significantly lower in the tumor group than in the control group, suggesting that it may be due to excessive consumption by tumor growth. N(omega)-Hydroxyarginine serves as a precursor in nitric oxide synthesis, while carbon monoxide is closely associated with tumor progression (Reddy et al., 2022). Consequently, these differential metabolites may assist in identifying LM in lung cancer.

### Cross-correlation analysis between the microbiota and metabolites

**Figure 8:**
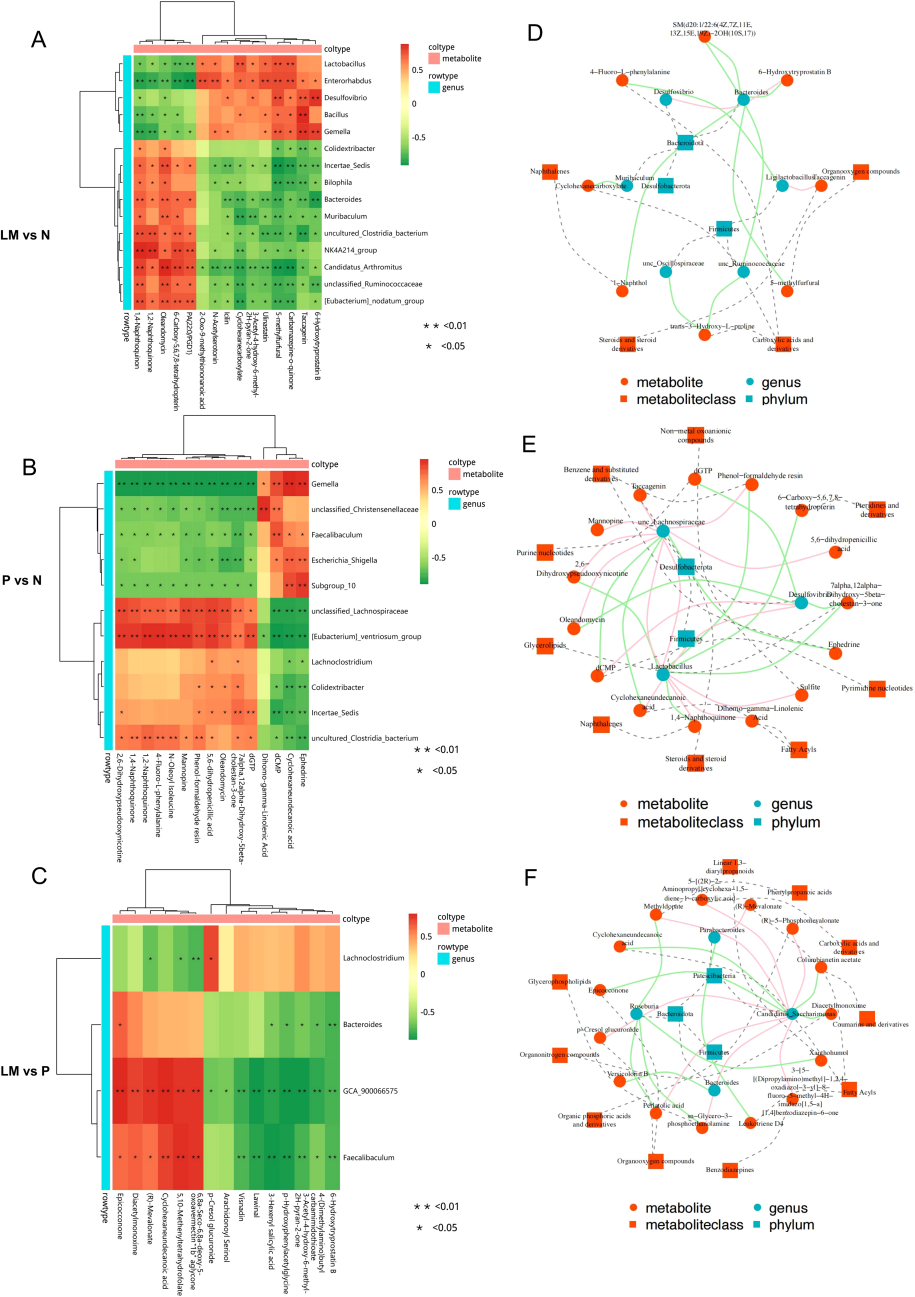
Cross-correlation analysis between microbiota and metabolites. **(A-C)** Heat map of correlation analysis between metabolites and microorganisms. **(D-F)** Network diagram illustrating the investigation of metabolite-microbe associations. The solid line illustrates the association between metabolites and microbial genera, with positive correlations depicted in red and negative correlations in green. The dashed line delineates the categorization of metabolites and microorganism. *p < 0.05, **p < 0.01.

Conduct a correlation analysis between the microbiome and metabolome results to determine the presence of any correlation between the two variables. The association between species diversity and metabolites in the sample was assessed using the Pearson correlation coefficient. Identify the top 15 differential metabolites and microorganisms according to the absolute value of log2FC, and subsequently create a correlation heatmap, as illustrated in **Figures 8A-C**. In comparison to the N group, the LM group exhibited significant variations in microbial communities, including Bacteroides, Muribaculum, and unclassified Ruminococcaceae. These communities were positively correlated with the metabolite oleandomycin and negatively correlated with 6-hydroxytryprotatin B and 5-methylfurfur. Desulfovibrio exhibits a positive correlation with 6-Hydroxytryprotatin B. In contrast to the subcutaneous tumor group, the differential metabolite N-acetylseletonin in the leptomeningeal metastatic group exhibits a positive correlation with the microorganism Eubacteria. Lachnoclostridium is associated with several metabolites, including 6-Hydroxytryprotatin B, 3-Hexenyl salicylic acid, and 5,10-Methenyltetrahydrofolate.

A correlation network diagram was plotted, with red lines representing positive correlations, green lines indicating negative correlations, and dashed lines denoting the classification of metabolites and microorganisms **Figures 8D-F**.

## Discussion

The findings revealed distinct differences in the gut microbiota composition and metabolite profiles among lung adenocarcinoma patients with leptomeningeal metastasis (LM), those with subcutaneous metastasis (P), and those in the wild-type group (N). By integrating dual-omics approaches, the underlying pathogenesis was investigated and potential biomarkers associated with leptomeningeal metastasis were identified in this study.

In terms of microbial diversity, the P and LM groups presented marginally greater species richness than did the N group. At the phylum level, Bacteroidetes was the most abundant genera across all three cohorts, followed by Firmicutes. These two phyla constitute the core microbial communities in the gut, playing pivotal roles in maintaining intestinal homeostasis, and their dysregulation has been linked to numerous diseases (Abdallah Ismail et al., 2011; Lin et al., 2017). Previous studies have demonstrated that alterations in the Firmicutes-to-Bacteroidetes (F/B) ratio are correlated with multiple pathological conditions. Elevated F/B ratios are frequently associated with metabolic disorders such as obesity and hypertension (Magne et al., 2020; Moon et al., 2020), whereas Firmicutes contribute to intestinal barrier stabilization through short-chain fatty acid (SCFA) synthesis (Mantovani et al., 2024). Conversely, reduced F/B ratios may compromise barrier integrity and exacerbate inflammatory responses as observed in inflammatory bowel disease (Yan et al., 2022).

Notably, the LM group presented a significantly greater F/B ratio than both the P and N groups did—a finding that contradicts previous reports on breast cancer (An et al., 2023). We propose that metabolites derived from Firmicutes, particularly butyrate, may exert tumor-suppressive effects. Consequently, the tumor microenvironment might trigger a compensatory increase in Firmicutes abundance to counteract malignant progression. Additionally, the increase in the F/B ratio in this study was primarily attributed to a significant reduction in Bacteroidetes in the LM group. A study investigating gut microbiota distribution in normal and anxious populations revealed a significant association between decreased Bacteroidetes abundance and anxious mood (Mason et al., 2020). Therefore, we presume that the establishment of the LM model induces anxious behavior in mice, which aligns with the observed reduction in their daily activity.

Alpha diversity exhibited marginal differences among the three groups; however, these differences lacked statistical significance (p > 0.05). This metric primarily reflects species richness (e.g., the Chao1 index) and evenness (e.g., the Shannon index). The lack of statistical significance in alpha diversity is likely attributed to two primary factors. First, the relatively small sample size of mice in this experiment may limit the power. On the other hand, the lack of mature T-cell immunity in nude mice may lead to insufficient activation of tumor-induced immune-inflammatory pathways, thereby weakening the regulatory effect on the gut microbiota. In contrast, beta diversity, analyzed via principal coordinate analysis (PCoA) on the basis of Bray–Curtis dissimilarity, revealed distinct clustering patterns among the groups. The PCoA plot clearly revealed separation of the microbial communities across the three cohorts, with significant intergroup dissimilarity (p = 0.001), underscoring compositional divergence at the taxonomic level.

The Lactobacillaceae family exhibited significantly greater abundance in the LM and P groups than in the wild-type group. Lactobacillus (a genus within Lactobacillaceae) is a beneficial commensal known to inhibit pathogenic bacterial colonization, enhance mucosal immunity, and exert protective effects against colorectal carcinogenesis (Hendler and Zhang, 2018; Eslami et al., 2019; Han et al., 2021). A study on lung cancer indicated that a greater abundance of Lactobacillus in the oral cavity was significantly associated with a higher lung cancer risk (Hosgood et al., 2020), which aligns with the findings of this research.

Differentially abundant metabolite analysis revealed that, compared with the P group, the LM group presented significant alterations in metabolic pathways, including tryptophan metabolism, nicotinic acid and nicotinamide metabolism, and steroid hormone biosynthesis. The key enriched metabolites in these pathways included N-acetylserotonin (NAS), 6-hydroxymelatonin, xanthurenic acid, 6-hydroxypseudooxynicotine, and β-D-ribosylnicotinate.

NAS, the direct biosynthetic precursor of melatonin, has been implicated in cancer progression. Studies have demonstrated that an elevated NAS/melatonin ratio promotes breast cancer cell survival and metastasis by mimicking brain-derived neurotrophic factor (BDNF) through TrkB receptor activation (Jang et al., 2010; Anderson, 2019a; Anderson, 2019b). This mechanism is particularly relevant in HER2+ breast cancers, where BDNF-mediated phosphorylation of TrkB-HER2 heterodimers potentiates brain metastasis (Choy et al., 2017). A clinical study has shown that NSCLC patients with increased BDNF/TrkB expression in tumor tissues have significantly shorter survival than those with low expression (Okamura et al., 2012). Additionally, research indicates that high BDNF/TrkB expression in NSCLC is associated with lymph node metastasis and vascular invasion (Zhang et al., 2010). Furthermore, patients with increased BDNF levels in primary lung adenocarcinoma might have a higher risk of developing brain metastasis, and central nervous system metastasis showed an elevated expression of BDNF compared to their matched primary lesions. The results showed that BDNF might drive an immunosuppressive tumor microenvironment (TME) by reeducation of tumour-associated macrophages (TAMs) toward a pro-tumorigenic M2 phenotype, particularly in brain metastasis (Yu et al., 2022). These findings position NAS as a potential biomarker for LM risk stratification in NSCLC, although further validation is needed to elucidate its precise role in lung cancer pathogenesis.

Xanthurenic acid has been implicated in intercellular signaling within the brain, although its role in oncogenesis remains poorly characterized (Maître et al., 2024). The elevated levels observed in the LM cohort may reflect tumor-host metabolic crosstalk, potentially driven by leptomeningeal tumor cells modulating neurotransmitter-like signaling pathways.

β-D-Ribosylnicotinate, a niacin derivative, serves as an intermediate in the conversion of niacin to nicotinamide—a critical precursor for nicotinamide adenine dinucleotide (NAD+) biosynthesis via the salvage pathway. This pathway is governed by nicotinamide phosphoribosyltransferase (NAMPT), an enzyme that is overexpressed in diverse malignancies, including neuroendocrine tumors, lymphomas, and adrenocortical carcinomas (Kozako et al., 2019; Sawicka-Gutaj et al., 2022; Nomura et al., 2023). Preclinical evidence has demonstrated that NAMPT inhibition induces rapid cytotoxicity in small cell lung cancer (Nomura et al., 2023). The significant accumulation of β-D-ribosylnicotinate in leptomeningeal metastases raises a critical hypothesis; pharmacologically limiting its biosynthesis may disrupt NAD+-dependent tumor cell survival mechanisms. This therapeutic strategy warrants systematic exploration in NSCLC-associated LM models.

Correlation analysis revealed a significant positive association between the abundance of the metabolite NAS and that of the gut microbiota genus Eubacterium (P = 0.03). Notably, Eubacterium has been identified as a proinflammatory pathobiont capable of promoting colitis-associated colorectal carcinogenesis via NF-κB activation (Wang et al., 2021). Research indicates that TrkB participates in NF-κB signaling via the ARMS protein subsequent to its interaction with BDNF (Sniderhan et al., 2008). We hypothesize that NAS and Eubacterium may cooperatively amplify NF-κB-driven transcriptional programs, fostering a protumorigenic microenvironment through dual mechanisms.

A comparative analysis of the gut microbiota and metabolites between subcutaneous lung cancer xenografts and LM models was conducted in this study. To establish a reproducible LM model, fecal samples from nude mice were prioritized for sequencing because of their utility in controlled experimental settings. While environmental variables could be further standardized, murine-derived sequencing data inherently lack direct translational equivalence to human pathophysiology. The biological and clinical relevance of the identified microbial and metabolic signatures necessitates functional validation in patient cohorts and mechanistic studies to confirm their role in LM pathogenesis.

## Conclusion

This study employed 16S rRNA sequencing and LC/MS to profile gut microbiota and fecal metabolites in nude mouse models of lung adenocarcinoma. Comparative analysis revealed significant divergence in microbial composition and metabolic profiles in the LM group relative to both N and P cohorts. Correlation networks demonstrated associations between specific microbial taxa (e.g., Eubacterium) and dysregulated metabolites (e.g., N-acetylserotonin, β-D-ribosylnicotinate), suggesting microbiota-metabolite crosstalk in LM pathogenesis. Targeted modulation of these microbial and metabolic signatures could unveil novel therapeutic strategies for LM prevention, early diagnosis, and precision treatment, addressing a critical unmet need in lung oncology.

## Data Availability

The datasets given in this work are available in internet sources. The names of the repositories and accession number(s) are provided below: https://dataview.ncbi.nlm.nih.gov/object/PRJNA1245166?reviewer=4q5h473vvp2gsuo863rakj2nj6. Sequencing data can be accessed at NCBI SRA BioProject, accession No. PRJNA1245166, and is publicly available as of the date of publication.
